# ITGB2 related to immune cell infiltration as a potential therapeutic target of inflammatory bowel disease using bioinformatics and functional research

**DOI:** 10.1111/jcmm.18501

**Published:** 2024-08-01

**Authors:** Rong Xu, Wei Du, Qinglong Yang, Ashuai Du

**Affiliations:** ^1^ Department of Pathology, Changde Hospital, Xiangya School of Medicine Central South University (The First People's Hospital of Changde City) Changde Hunan China; ^2^ Department of General Surgery Guizhou Provincial People's Hospital Guiyang Guizhou China; ^3^ Department of Infectious Diseases Guizhou Provincial People's Hospital Guiyang Guizhou China

**Keywords:** bioinformatics analysis, immune cell infiltration, inflammatory bowel disease, STAT1;ITGB2

## Abstract

Inflammatory bowel disease (IBD) is a chronic systemic inflammatory condition regarded as a major risk factor for colitis‐associated cancer. However, the underlying mechanisms of IBD remain unclear. First, five GSE data sets available in GEO were used to perform ‘batch correction’ and Robust Rank Aggregation (RRA) to identify differentially expressed genes (DEGs). Candidate molecules were identified using CytoHubba, and their diagnostic effectiveness was predicted. The CIBERSORT algorithm evaluated the immune cell infiltration in the intestinal epithelial tissues of patients with IBD and controls. Immune cell infiltration in the IBD and control groups was determined using the least absolute shrinkage selection operator algorithm and Cox regression analysis. Finally, a total of 51 DEGs were screened, and nine hub genes were identified using CytoHubba and Cytoscape. GSE87466 and GSE193677 were used as extra data set to validate the expression of the nine hub genes. CD4‐naïve T cells, gamma–delta T cells, M1 macrophages and resting dendritic cells (DCs) are the main immune cell infiltrates in patients with IBD. Signal transducer and activator of transcription 1, CCR5 and integrin subunit beta 2 (ITGB2) were significantly upregulated in the IBD mouse model, and suppression of ITGB2 expression alleviated IBD inflammation in mice. Additionally, the expression of ITGB2 was upregulated in IBD‐associated colorectal cancer (CRC). The silence of ITGB2 suppressed cell proliferation and tumour growth in vitro and in vivo. ITGB2 resting DCs may provide a therapeutic strategy for IBD, and ITGB2 may be a potential diagnostic marker for IBD‐associated CRC.

## 1 INTRODUCTION

Inflammatory bowel disease (IBD), a chronic relapsing–remitting gastrointestinal systemic disease characterized by inflammatory processes requiring lifelong treatment,^1^ includes Crohn's disease (CD) and ulcerative colitis (UC).^2^, ^3^ Intestinal mucosal inflammation in IBD manifests as abdominal pain, diarrhoea, bloody stools, emaciation and a series of inflammatory reactions, including infiltration of neutrophils and macrophages, resulting in the generation of large amounts of cytokines, proteolytic enzymes and free radicals, which damage the structure of the intestinal mucosa.^4^, ^5^

As a global disease, the incidence and prevalence of IBD are rapidly increasing and have attracted considerable research interest.^6^, ^7^, ^8^ The aetiology and pathogenesis of IBD are complex and have not yet been elucidated. Nevertheless, studies have reported that the pathogenesis of IBD is associated with host genetics, immunity, the intestinal microbiome and environmental factors.^9^, ^10^, ^11^ Therefore, improving our understanding of the mechanisms involved in IBD and exploring diagnostic biomarkers are crucial for studying IBD. Here, we conducted bioinformatics analysis to explore the potential mechanisms involved in IBD and identify novel targets for use in future clinical research.

IBD is an immunological inflammatory disease^12^, ^13^ induced by dysfunction of the immune system in the intestine. Immune cells (T cells, dendritic cells [DCs], macrophages, B cells, NK cells and neutrophils) regulate intestinal homeostasis.^14^ During the development of IBD, a combination of genetic and environmental factors trigger IBD‐initiating events, resulting in intestinal mucosal barrier injury. Subsequently, innate immunity is activated, which impairs intestinal mucosa homeostasis by producing cytokines and chemokines, recruiting adaptive immune cells and inducing ‘cytokine storms.’ This ultimately leads to long‐term gut inflammation.^15^

**FIGURE 1:**
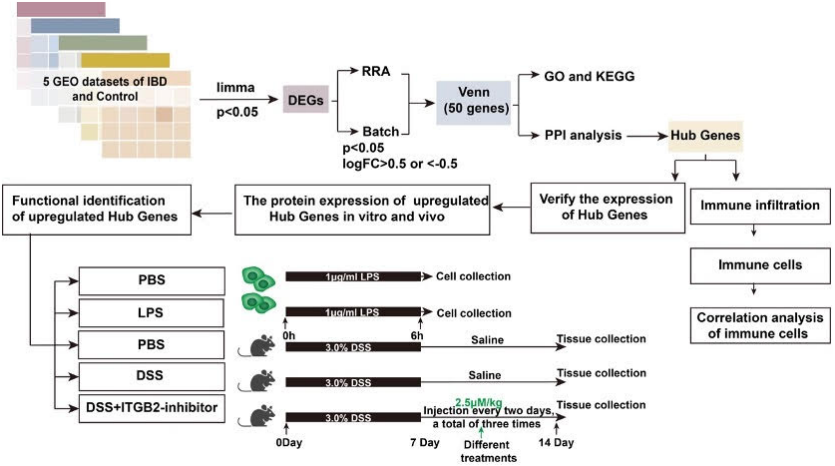
Flow chart of the study process for this research. Five GSE data sets were used for bioinformatics analysis; RRA and Batch were used to screen differential genes; Then, GO and KEGG were used to identify the potential function of these differential genes; PPI was to identify the hub genes, and hub genes verification were performed by in vitro and in vivo experiments. Furthermore, we applied CIBERSORT algorithm to evaluate the distribution of infiltration of immune cells in IBD and healthy control tissues. GO, Gene Ontology; PPI, protein–protein interaction; RRA, Robust Rank Aggregation.

Our study used CIBERSORT to analyse immune cell infiltration in IBD, and least absolute shrinkage selection operator (LASSO) regression and Wilcoxon tests to identify significantly different immune cell infiltration in IBD. We explored hub genes using two bioinformatics methods (Robust Rank Aggregation [RRA] and batch) and analysed the correlation between four significantly different types of infiltrated immune cells. We found that signal transducer and activator of transcription 1 (STAT1) and integrin subunit beta 2 (ITGB2) were significantly related to infiltrated immune cells. STAT1 has been reported to regulate LCP2 and TNFAIP2 to induce IBD,^16^ regulate the lineage commitment of Th1 and Th17 cells early, and maintain immunological functions in vitro and in vivo.^17^ However, the function of ITGB2 in IBD remains unclear. ITGB2 is an inflammatory and immune‐related gene that participates in the inflammatory response and inflammatory tumour transformation during IBD carcinogenesis.^18^, ^19^ Another study reported that ITGB2 is closely associated with EMT in colorectal cancer (CRC).^20^ IBD is closely associated with CRC, and patients with IBD are at higher risk of developing CRC.^21^ Therefore, the control of inflammation may contribute to the prevention and control of CRC. Herein, we tested the expression and function of ITGB2 in vitro and in vivo and measured the protein levels of ITGB2 in colitis‐associated cancer (CAC) tissues, which may provide new therapeutic approaches for IBD and novel strategies for preventing IBD‐associated CRC. A flowchart of the study process is shown in Figure 1.

## 2 METHOD AND MATERIALS

### 2.1 Data Collection and preprocessing

The GEO dataset was retrieved from the GEO database (http://www.ncbi.nlm.nih.gov/geo)database. Raw data were retrieved from five test expression profiling arrays (GSE112366, GSE179285, GSE186582, GSE207022 and GSE24287).

### 2.2 Differentially expressed genes (DEGs) analysis

The ‘batch correction’ was used for batch correction, normalization of the raw datasets, and analysis of DEGs. ‘RRA’ was used to merge the raw datasets from five GSE datasets into a gene matrix and calculate the DEGs using the ‘limma’ package as described previously.^22^ A Venn diagram was used to intersect the results of the two methods and identify accurate candidate differential genes. The filter criteria for DEGs were *p*‐value <0.05 and log FC >1 or < −1.

### 2.3 Principal Component Analysis (PCA)

PCA was used to analyse the intragroup repeatability of the five GSE datasets before and after batch correction. The immune cell clustering analysis of the control and IBD groups was performed using PCA. The results and statistical analysis were shown using the R package ‘ggplot2.’

### 2.4 Functional enrichment analysis

Gene Ontology (GO) was used to identify the significantly different GO terms using the ‘clusterProfilter’ package. *p* < 0.05 was considered the threshold for significantly enriched functional evaluation.

### 2.5 Screening of Hub Genes

The protein–protein interaction (PPI) network was constructed using the online website package STRING (https://string‐db.org). The PPI network of the DEGs was visualized using Cytoscape (v 3.7.2), and CytoHubba was used to score each node gene using MCC, DMNC, MNC, Degree, BottleNeck, EcCentricity, Closeness, Radiality, Betweenness and Stress. Node genes calculated by a top‐10 algorithm were then used to obtain hub genes using the ‘UpSet’ package. The GSE207022 dataset was used as the test dataset to verify the hub genes.

### 2.6 ROC analysis

The area under the receiver operating characteristic (ROC) curve (AUC) was used to identify the effectiveness of the discriminative diagnosis of IBD from control samples in the GSE207022, GSE87466 and GSE126124 dataset.

### 2.7 Distribution of Immune cell subtype

The CIBERSORT algorithm was used to analyse the distribution of immune cell infiltration in patients with IBD and controls. The CIBERSORT website is an analytical tool that can estimate the abundance of cell types based on gene expression data, which can infer 22 cell types using LM22. The data with *p*‐value <0.05 was retained for the following visualization using ‘glmnet,’ ‘vioplot,’ ‘ggplot,’ and ‘corplot.’ Moreover, LASSO Cox regression and the Wilcoxon test were utilized to analyse the significantly different infiltrates of immune cells in IBD using ‘glmnet.’

### 2.8 Cell culture and tissue collection

Caco2 cells were used as an IBD cell model, as previously described.^23^ The cells were cultured in 5% CO_2_ at 37°C. High‐glucose Dulbecco's modified Eagle's medium (DMEM) supplemented with 10% foetal bovine serum was used as the complete medium for Caco2. The ITGB2 siRNA was purchased from RIBOBIO (Guangzhou, China). Lipofectamine 2000 (Life Technologies, Carlsbad, CA, USA) was used for siRNA transfection according to the manufacturer's instructions. For the cell IBD model, Caco2 cells were cultured with medium containing lipopolysaccharide (LPS) (1 μg/mL) (Sigma‐Aldrich, Burlington, MA, USA) at different time points.

CRC and matched adjacent normal tissues were collected from Guizhou Provincial People's Hospital (Guizhou, China). The Ethics Committee of Guizhou Provincial People's Hospital approved all sample collection procedures.

### 2.9 qPCR and western blot analysis

The qPCR assay was performed as previously described.^24^ After quantification, total RNA was extracted and reverse‐transcribed into cDNA using a NanoDrop spectrophotometer. The gene mRNA level was detected using MonAmp™ SYBR® Green qPCR Mix in ABI 7500 and was calculated using the 2^−DDCt^ method. Tsingke Biotechnology Co., Ltd. (Beijing, China) synthesized primers for these genes, and the primer sequences are listed in Table S1.

Western blotting was performed as previously described.^25^ Briefly, cells were lysed in a strong RIPA buffer‐added protease inhibitor (100x), denatured at 100°C for ten minutes, then resolved by SDS‐page. Tissues were pulverized in liquid nitrogen, and total protein was extracted. Primary antibodies against STAT1 (R25801), CCR5 (252550) and ITGB2(R381795) were purchased from Zen Biosciences (Chengdu, China). Primary antibodies against GAPDH (GB15002‐100), horseradish peroxidase (HRP)‐conjugated goat anti‐mouse IgG (H + L) (GB23301) and HRP‐conjugated Rabbit Anti‐Goat IgG (H + L) (GB23204) were purchased from Servicebio (Wuhan, China). The bands were reacted with the HPR substrate, and chemiluminescence images were captured using a Tanon 5200Multi (Tanon, Shanghai, China) Clone formation and CCK8 assay. Exponentially growing cells were added to 6‐well plates at a density of 1 × 10^3^ cells/well. About 10 days later, the colonies were treated with crystal violet and washed with water. Clones with more than 50 cells were counted. For the CCK8 assay, we seeded 2 × 10^3^ cells into 96‐well culture plates. OD values were tested at 24 h, 48 h, 72 h and 96 h.

### 2.10 In vivo experiments

Six‐week‐old Balb/c female mice were purchased from the Animal Department of Central South University, and all experiments were performed with the approval of the Central South University of Science and Technology Animal Care and Use Committee. Mice with colitis were established as previously described.^23^ Dextran sulphate sodium salt (DSS) was purchased from MERCK (Rahway, NJ, USA). For ITGB2 silencing experiments, mice were injected peritoneally with 20 nM in vivo siRNA (RIBOBIO, Guangzhou, China) three times. The mice were sacrificed 14 days after DSS treatment. For the tumour transplantation model, 5 × 10^6^ cells were injected into Balb/c nude mice (5 weeks old) on the right flanks. Mice were injected peritoneally with 20 nM ITGB2 siRNA, and subsequent tumour volumes and weights were measured. As previously described, haematoxylin and eosin (H&E) staining was used to detect morphological changes between the IBD and PBS groups.^26^ Two pathologists evaluated the pathological features.

### 2.11 Immunohistochemistry

Samples were fixed in 10% neutral buffered formalin and the paraffin embedded biopsies sections were subjected to immunofluorescence and immunohistochemistry staining. Primary antibody against ITGB2 (1:50, R381795, Zen Biosciences) or Ki67 (1:100, ab15580, abcam). Then, the slides were incubated with secondary antibodies and stained with the DNB staining kit (TT‐0805, MXB, biotechnologies).

### 2.12 ELISA

A Mouse IL‐6 ELISA Kit (PI326), Mouse IL‐1β ELISA Kit (PI301) and Mouse TNF‐α ELISA Kit (PI512) from Beyotime (Shanghai, China) were used to quantify the IL‐6, IL‐1β and TNF‐α levels in mouse serum. To ensure consistency, the evaluation was conducted five times for each group.

### 2.13 Statistical analysis

R software (version 4.0.3) was used for image visualization and statistical analysis. Spearman's correlation analysis analysed the correlation between hub gene expression and the differentially expressed immune cells. ROC curve analysis was performed to evaluate the diagnosis of hub genes in patients with IBD. A Student's *t*‐test or one‐way analysis of variance (ANOVA) was used for statistical analyses between two or more groups.

## 3 RESULTS

### 3.1 Identifications of DEGs between IBD and control tissue

**FIGURE 2:**
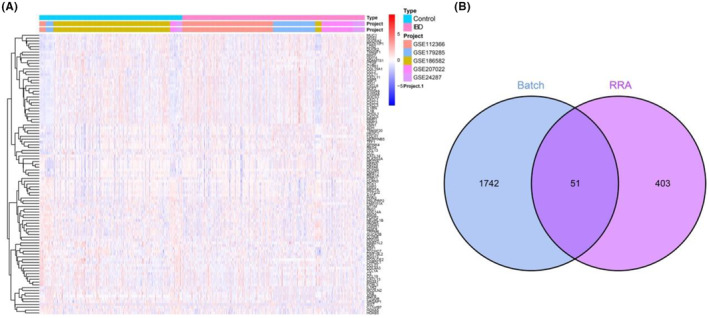
Identification of DEGs. (A) 78 differential DEGs were visualized in a heatmap determined by ‘Batch.’ (B) Venn diagram was used for DEGs intersection screened from the two methods. DEGs, differentially expressed genes.

This study used five GEO datasets to identify candidate genes involved in IBD, and 51 DEGs were explored and applied in subsequent analyses. First, the raw data were batch‐corrected and normalized (Figure S1), and 1793 DEGs were identified. Another 454 DEGs were identified using the RRA method, and a heat map and Venn diagram were constructed to visualize the final DEGs (Figure 2A,B).

### 3.2 GO and KEGG Analysis

**FIGURE 3:**
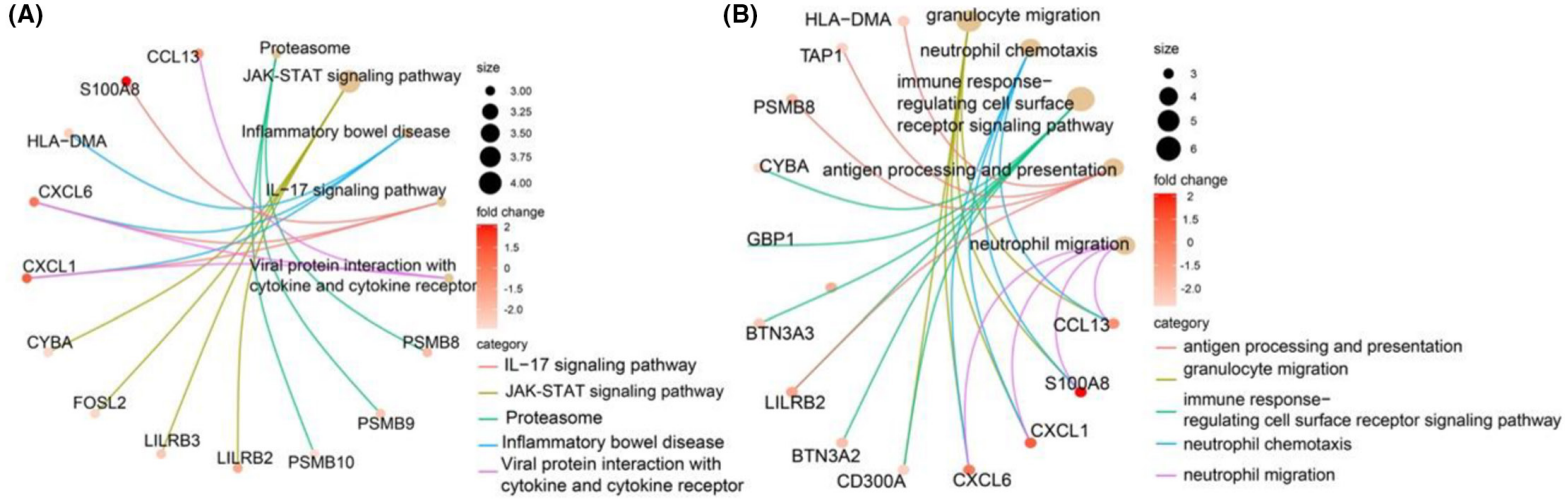
Functional analysis. (A)The results of KEGG as shown by cneplot. (B) The results of GO as shown in cneplot. GO, Gene Ontology.

To analyse the DEGs' functional role and signalling pathways, we conducted a KEGG analysis to investigate the signalling pathways in which the DEGs participated. The centre plot shows the functional enrichment of the DEGs by GO analysis. As shown in Figure 3A, the JAK–STAT signalling pathway accounted for most of the DEGs, followed by the proteasome and IL‐17 signalling pathways. GO analysis showed that the DEGs were mainly related to immune‐related processes or responses (Figure 3B). Overall, our study found that DEGs were associated with immune cells.

### 3.3 Hub Genes validation

**FIGURE 4:**
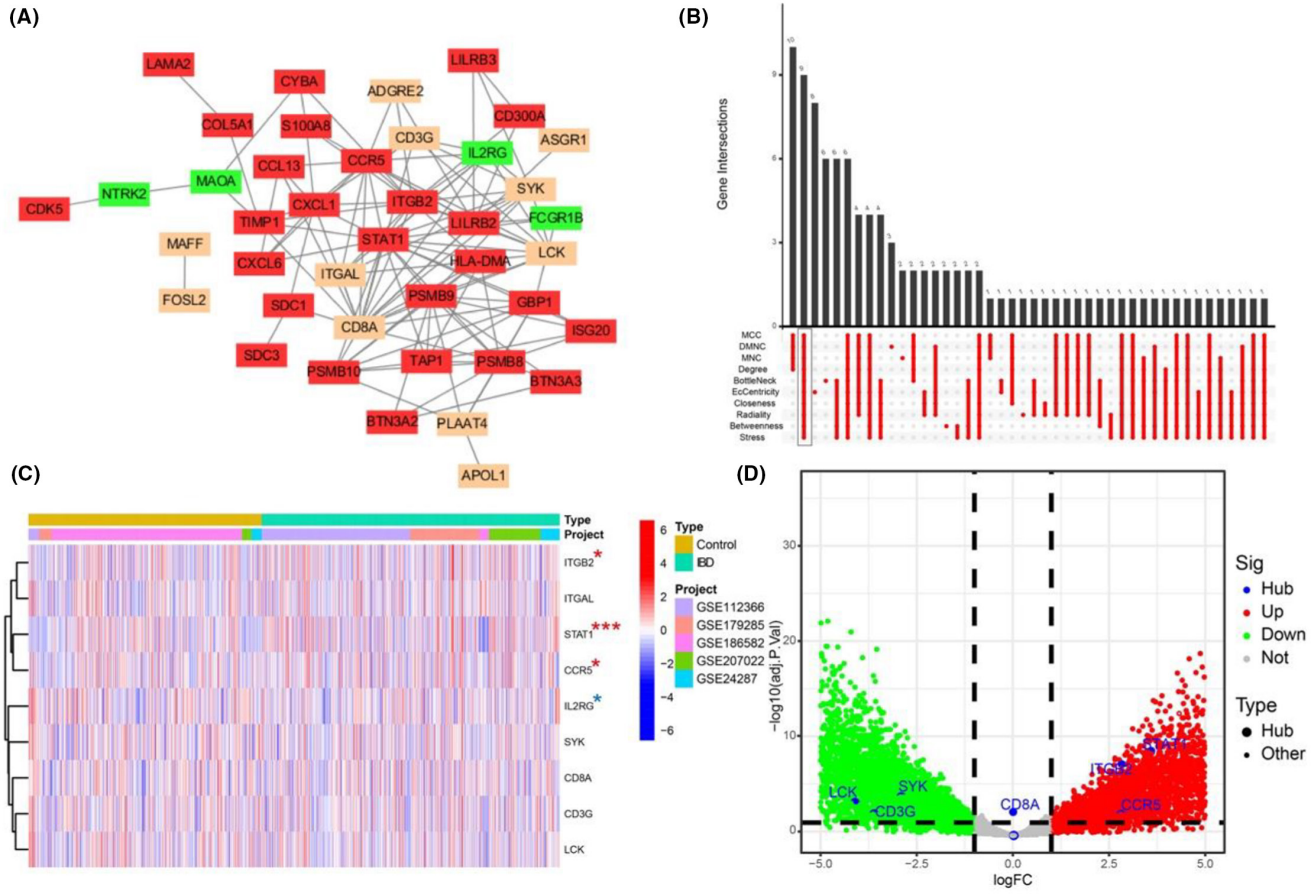
Construction of PPI network for differentially expressed genes. (A) Visualization of PPI network using Cytoscape software. Nodes in red represent upregulated in IBD, nodes in green indicate no significantly difference between IBD and control, and nodes in orange represent genes downregulated in IBD. (B) Nine hub genes were screened through ten algorithms. (C, D) The expression of nine hub genes shown in a heatmap and volcano map. DEGs, differentially expressed genes; IBD, inflammatory bowel disease; PPI, protein–protein interaction.

**FIGURE 5:**
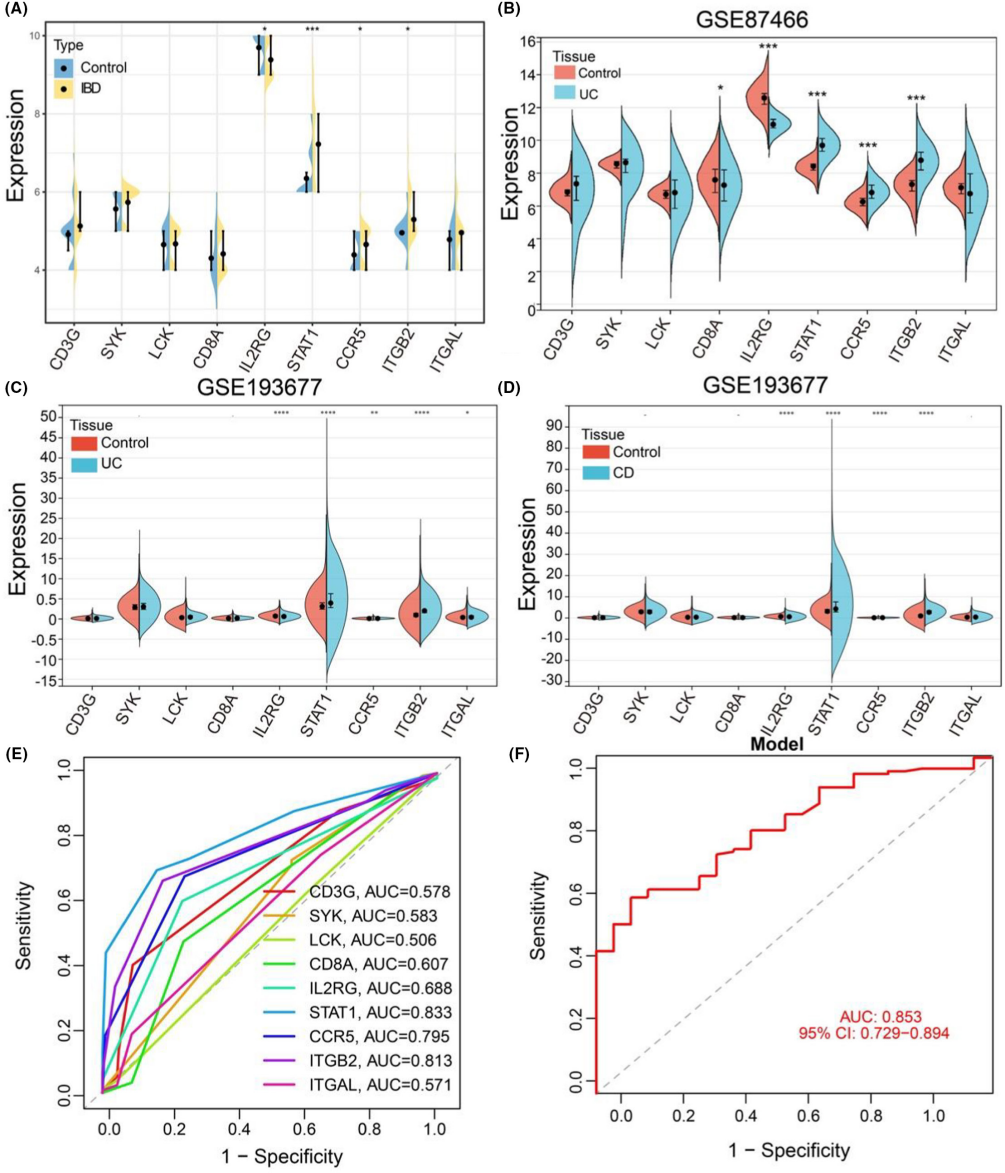
Validation of hub genes (A) The expression of nine hub genes in in GSE207022. (B) GSE87466 was used for validating the expression of nine hub genes. (C, D) GSE193677 was used to analysis the expression of nine hub genes. (E) ROC analysis was used to evaluate the diagnostic effectiveness of hub genes for IBD in validation GSE87466. (F) Combined AUC of STAT1 and ITGB2 in validation GSE87466. AUC, area under the curve; IBD, inflammatory bowel disease; ROC, receiver operating characteristic.

First, the PPI network of DEGs was constructed using STRING (Figure 4A), where the nodes in red represent upregulated genes, those in orange represent downregulated genes, and those in green represent genes showing no significant difference in expression between IBD and control tissues. We screened nine hub genes (ITGB2, ITGAL, STAT1, CCR5, IL2RG, SYK, CD8A, CD3G and LCK) using the ‘UpSet’ package and marked them with boxes in Figure 4B. The expression of the nine hub genes was visualized using a heat map of the merged GEO data (Figure 4C). Next, the expression levels of hub genes are presented in a volcano plot in the merged GEO data (Figure 4D). Data from GSE207022 showed that the expression levels of STAT1, CCR5 and ITGB2 were significantly higher in IBD (*p* < 0.05) (Figure 5A). GSE87466 was used for further validation, as shown in Figure 5B, and the expression of STAT1, CCR5 and ITGB2 was significantly increased in UC, which is consistent with the above results for GSE207022. Additionally, we screened the expression of hub genes in another GSE database that contained 872 UC samples, 1157 CD samples and 461 controls. The results showed that STAT1, CCR5 and ITGB2 were significantly upregulated in UC and CD, while IL2RG was downregulated in UC and CD compared to the control groups (Figure 5C,D). Therefore, we evaluated the diagnostic effectiveness of hub genes for IBD using ROC curve analysis. Generally, an AUC of more than 0.8 was considered sufficient for diagnosing disease with excellent specificity and sensitivity. STAT1 had the highest AUC value, followed by ITGB2 and CCR5 (Figure 5E). The combined AUC of STAT1 and ITGB2 reached 0.853 (95% CI 0.729–0.894), indicating that STAT1 and ITGB2 can differentiate patients with IBD from controls with higher specificity and sensitivity (Figure 5F). Additionally, we analysed the diagnostic values of these two genes using extra datasets GSE87466 and GSE126124. The results showed that the combined AUC of STAT1 and ITGB2 reached 0.789 (95% CI 0.753–0.834) (Figure S2), indicating that STAT1 and ITGB2 can differentiate patients with IBD from controls with higher specificity and sensitivity.

### 3.4 Immune cell infiltration

**FIGURE 6:**
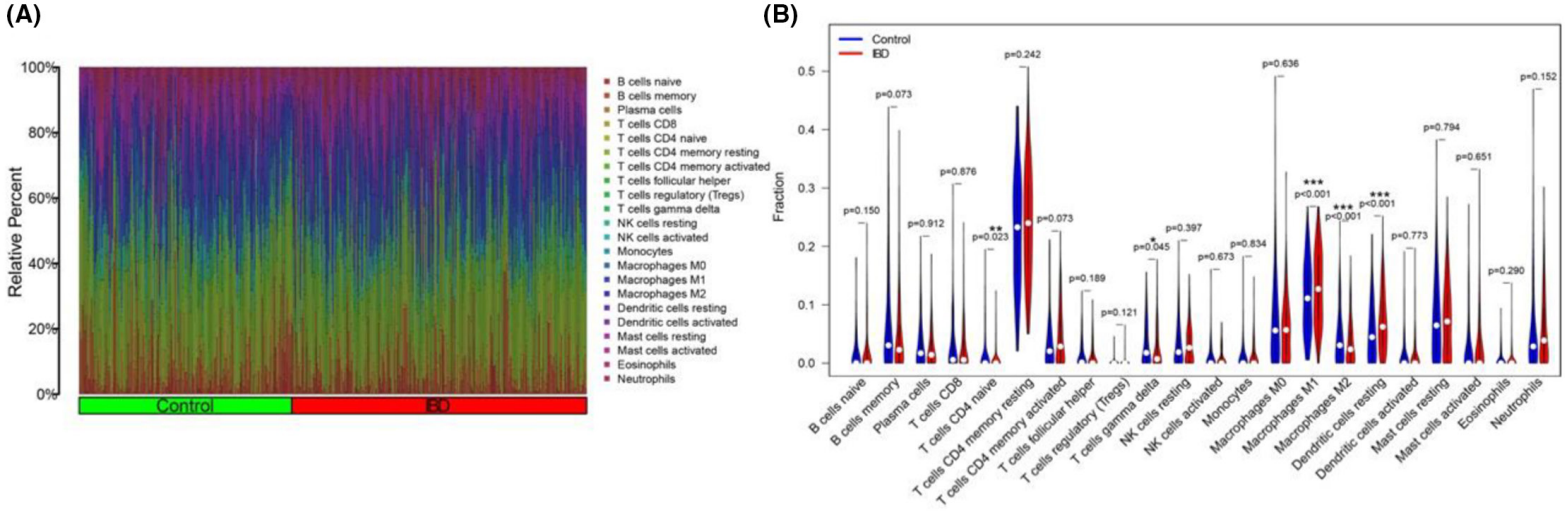
Immune cell infiltration in IBD and control tissues. (A) The 22 types of immune cells shown in a histogram. (B) Wilcoxon test was performed to identify the different immune cells in IBD and control. IBD, inflammatory bowel disease.

**FIGURE 7:**
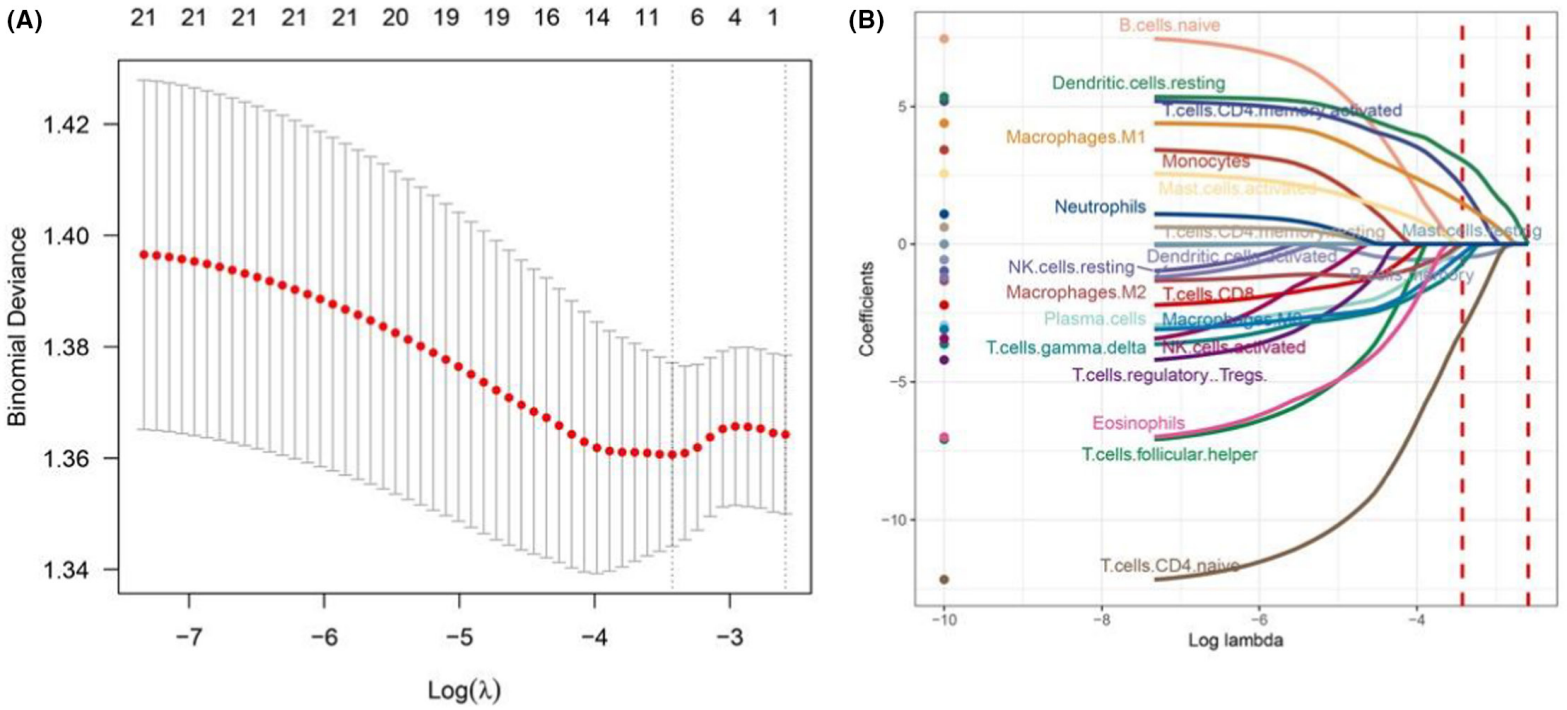
LASSO regression (A) and cvfit (B) were used to analyse the immune cell infiltration in IBD and control. IBD, inflammatory bowel disease; LASSO, least absolute shrinkage selection operator.

In total, 941 IBD and 107 control tissues from five GEO datasets were screened for immune cell infiltration. Figure 6A shows a histogram of the constitution of the 22 immune cell types in IBD and control tissues. Wilcoxon test was performed to identify the different immune cells in IBD and control tissues. (Figure 6B).The composition of the four intersecting immune cells (CD4‐naïve T cells, T cells, M1 macrophages and resting DCs) differed between the control and IBD groups. The distribution of the immune cells is presented as a heat map (Figure S3A). Moreover, we distinguished the IBD group from the control group using PCA, according to the proportion of immune cell infiltration in the intestinal mucosal tissue (Figure S3B). The Wilcoxon test and LASSO regression were also used to calculate the differences in immune cell infiltration (Figure 7A,B). CD4‐naïve T cells, gamma delta T cells, M1 macrophages and DCs were identified using the LASSO and Diff methods (Figure S3C). Compared with the control, M1 macrophages and resting DC levels were higher in IBD, whereas those CD4‐naïve T cells, gamma delta T cells and M2 macrophages were significantly lower. Moreover, the correlation between genes and immune cells was filtered by |R| > 0.4 and *p* < 0.05. The results showed that STAT1 and M1 macrophages were positively correlated (*R* = 0.53, *p* = 0.00055), and ITGB2 was positively correlated with DCs (*R* = 0.43, *p* = 0.0073) (Figure S3D).

### 3.5 Verification of the expression levels of STAT1, CCR5 and ITGB2 in vivo

**FIGURE 8:**
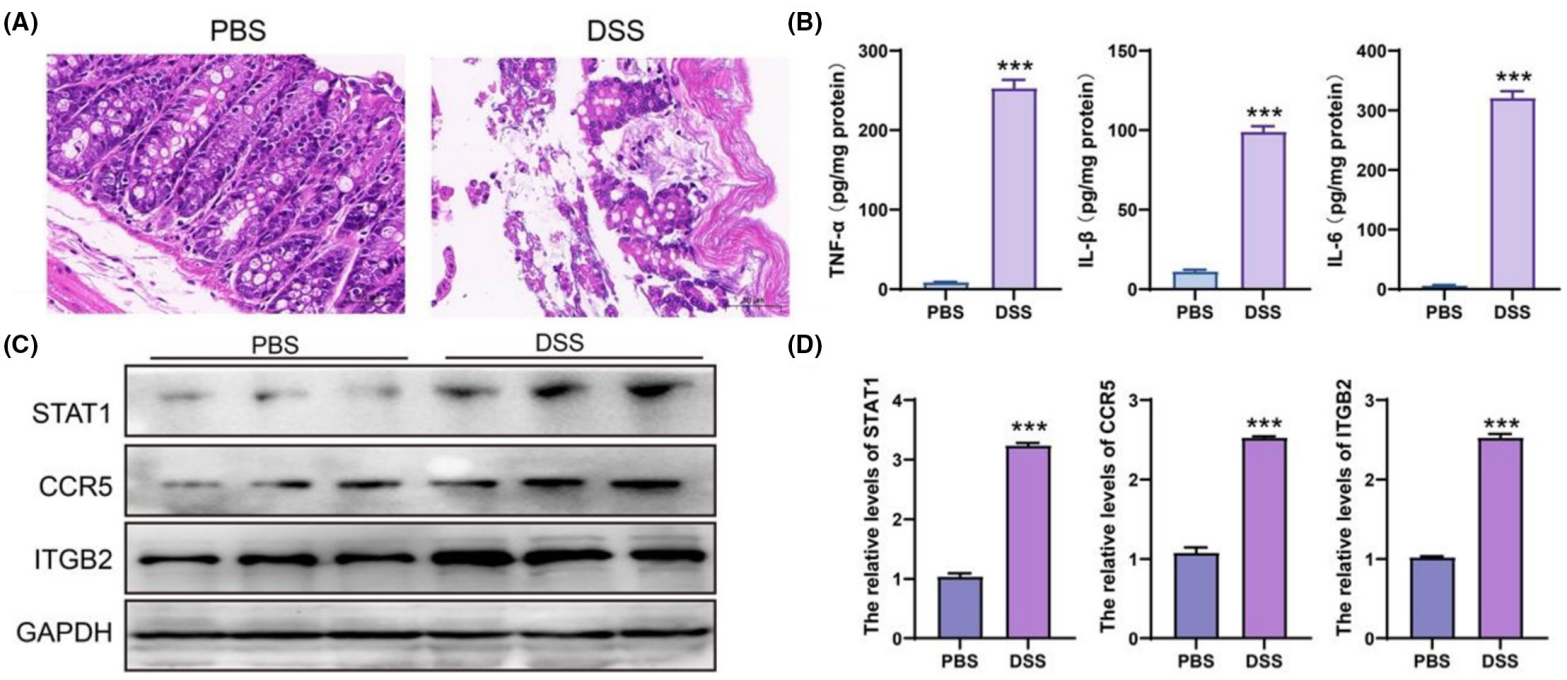
The expression of STAT1, CCR5 and ITGB2 in a mouse model of IBD. (A) HE assay was used to analyse the pathological changes in intestinal tissues in each group. (B) ELISA was used to evaluate the inflammatory cytokines in serum. (C, D) The protein level was detected by western blotting. ***p < 0.001, data represented as Mean ± SD. IBD, inflammatory bowel disease.

In this study, we selected three upregulated genes in patients with IBD for further verification. To better understand whether STAT1, CCR5 and ITGB2 levels were increased in IBD mice, IBD mice were established using DSS, as shown in Figure 8A. Serum IL‐6, IL‐1β, and TNF‐α levels were measured in each mouse (Figure 8B). In addition, western blotting results indicated that STAT1, CCR5 and ITGB2 protein levels significantly increased in the DSS‐treated mice (Figure 8C,D).

### 3.6 ITGB2 silencing ameliorated DSS‐induced IBD in mice

**FIGURE 9:**
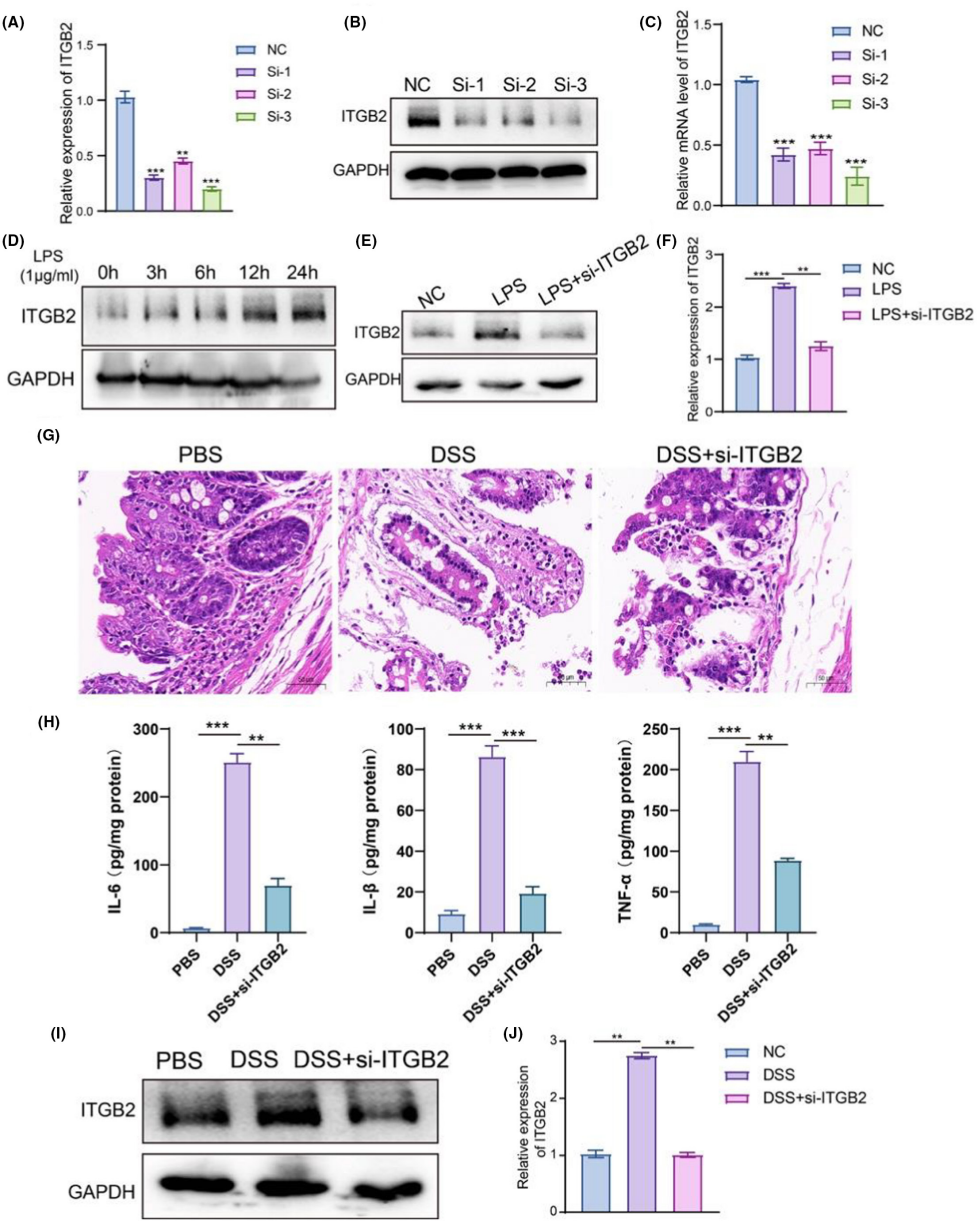
The effect of ITGB2 silence on DSS induced IBD mice. (A) qPCR was used to detect the expression level of ITGB2. (B, C) Western blotting was used to detect the protein level of ITGB2. (D)The protein level of ITGB2 was gradually increased over time under LPS treatment. (E, F) The protein level of ITGB2 in Caco2 cells. (G) The pathological changes in intestinal tissues in each group. (H) Inflammatory cytokines level was measured in each group. (I, J) Interference by ITGB2 significantly decreased the ITGB2 protein level in DSS‐induced IBD mice. **p < 0.01, ***p < 0.001, data represented as Mean ± SD. DSS, Dextran sulphate sodium salt; IBD, inflammatory bowel disease.

To explore the role of ITGB2 in the development of IBD, LPS, the most commonly used drug to stimulate inflammation in vitro, was used to establish an inflammation model.^27^, ^28^ First, we tested the three siRNA sequences of ITGB2 through qPCR and western blotting, and the results indicated that all three siRNA sequences demonstrated good silencing effects, with more than 50% interference (Figure 9A–C). Human intestinal Caco2 cells were exposed to LPS, and ITGB2 protein levels were measured at different time points using western blotting. ITGB2 protein levels gradually increased significantly after 6 h (Figure 9D). We performed rescue experiments to evaluate the role of ITGB2 in inflammation. After stimulation with LPS for 6 h, cells were transfected with 5 nM siRNA. ITGB2 silencing reverses the LPS‐induced increase in ITGB2 expression. Next, we treated IBD model mice with siRNA (20 nM per mouse) and evaluated the epithelial damage in mice compared with that in IBD mice (Figure 9E,F). HE staining results demonstrated that silencing ITGB2 ameliorated DSS‐induced IBD in mice. Silencing ITGB2 alleviated severe epithelial damage and decreased the serum levels of inflammatory factors (Figure 9G,H). Subsequently, we estimated the protein levels of ITGB2 in mice from the three groups, and the results indicated that ITGB2 expression significantly decreased in the DSS + si‐ITGB2 group (Figure 9I,J). Moreover, pan‐cancer analysis using TCGA datasets revealed that ITGB2 was significantly upregulated in several cancers (Figure S4A). Among these, high ITGB2 expression was associated with poor prognosis in CRC (Figure S4B). We collected 20 CAC and paracancerous tissue samples to identify the expression of ITGB2 further and analyse its correlation with clinical pathology. The results demonstrated that ITGB2 expression was significantly higher in CAC tissues than in the corresponding normal tissues (Figure S4C) and that ITGB2 expression was positively correlated with the TNM stage (*p* < 0.05) (Table S2). These results indicated that ITGB2 may drive IBD‐ and IBD‐associated CRC.

### 3.7 ITGB2 promote cell proliferation in vitro and in vivo

**FIGURE 10:**
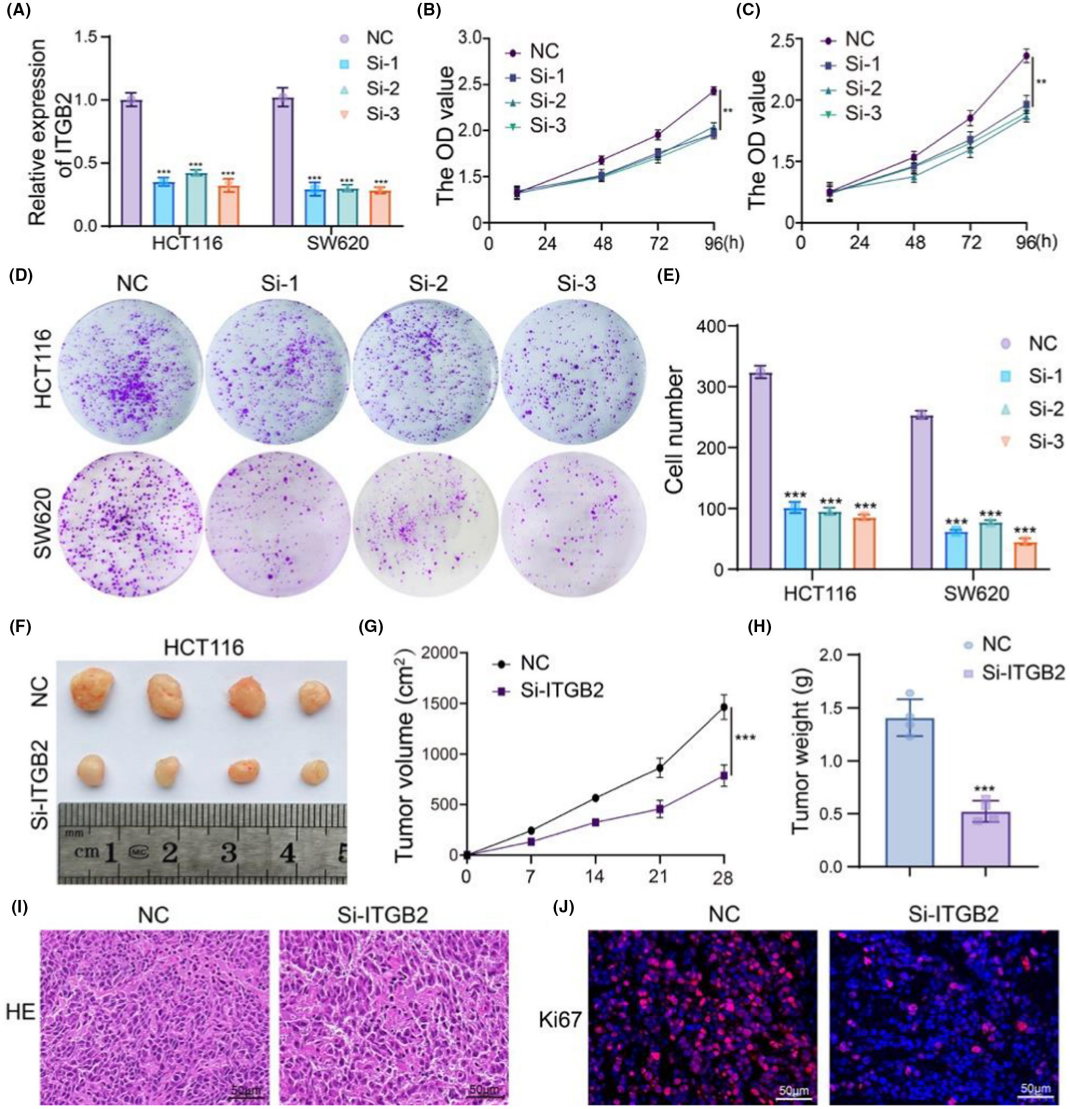
The silence ITGB2 could suppress cell growth in vitro and in vivo. (A) qPCR was used to detect the expression of ITGB2. (B, C)CCK8 assay was used to detect the cell viability. (D, E) Clone formation assay was used to detect the cell colony formation ability. (F) Pictures of xenograft tumours were shown in the ITGB2 silence group and control group (*n* = 4). (G) The growth curves of each group of xenograft tumours were displayed. (H) The xenograft tumour weight was measured and analysed. (I) HE assay was used to analysis the morphological changes in tumour. (J) immunofluorescence assay was used to detect the expression of ki67. **p < 0.01, ***p < 0.001, data represented as Mean ± SD.

To investigate this, we assessed the efficiency of siRNA sequences of ITGB2 in CRC cell lines (Figure 10A). CCK8 and clone formation assays showed that silencing ITGB2 suppressed cell proliferation and clone formation ability in HCT116 and SW620 (Figure 10B–E). Additionally, ITGB2 silencing inhibited tumour growth in vivo (Figure 10F) and decreased tumour volume (Figure 10G) and weight (Figure 10H). The HE assay showed that ITGB2 silencing decreased the number of tumour cells (Figure 10I). Immunofluorescence assays showed that silencing ITGB2 decreased the expression of Ki67 (Figure 10J). In conclusion, ITGB2 is a tumour‐promoting gene in CRC, and silencing ITGB2 may be a novel therapeutic strategy for CAC.

## 4 DISCUSSION

Inflammatory bowel disease is a lifelong, recurrent and remitting mucosal disease. Due to the long‐term disease process, patients suffer from inflammatory diseases to a large extent.^29^, ^30^ An increasing number of studies have shown that activation of infiltrating immune cells is responsible for developing IBD and sustained intestinal mucosal damage. Although the availability of various effective treatment options, including immunomodulators and biological agents, such as TNF and CAM inhibitors, has improved treatment efficacy, some patients do not respond or lose response to treatment over time.^31^, ^32^ Continuous disease monitoring, from symptom relief to endoscopic treatment, along with short‐ or long‐term treatment reactions, is crucial for providing tailored treatment algorithms for IBD patients.^15^ Therefore, exploring the immunopathogenesis of IBD and identifying new effective therapeutic targets is urgently required to develop novel models for IBD treatment.

The immune response caused by intestinal pathogenic infection is considered an important pathogenic factor disrupting the mucosal immune homeostasis and aggravating enteritis in IBD.^33^, ^34^ Thus, understanding the differences in the infiltrating immune cells between patients with IBD and healthy controls is conducive to developing novel therapeutic methods based on immune responses. For example, GPR174 induces IBD by regulating DCs to maintain intestinal homeostasis.^34^ Herein, CIBERSORT combined with the LASSO algorithm and Cox regression analysis were used to identify four significantly infiltrating immune cell types (CD4‐naïve T cells, gamma delta T cells, M1 macrophages and resting DCs). Compared with control tissues, M1 macrophages and resting DCs were significantly higher in IBD, whereas CD4‐naïve T cells, gamma delta T cells and M2 macrophages were significantly lower. In the inflammatory environment of the intestinal mucosa, M1 macrophages are the main type of macrophages that promote the inflammatory response.^35^ DCs facilitate immune activation during infection but exhibit lower antigen presentation in the resting state. In IBD, resting DCs are less capable of activating resting T cells, which accelerates inflammation.^36^ Thus, the results of our analysis are consistent with previous reports; our study highlights the importance of these immune cells in the pathogenesis of IBD through bioinformatic analysis.

The aetiology of IBD is related to dysregulation of key cellular genes.^37^, ^38^ Given the important functions of these immune cells and hub genes in IBD, the correlations between the nine candidate genes and the top four infiltrating immune cells were further evaluated. Furthermore, GSE87466 was used to validate the expression of hub genes and revealed that STAT1, CCR5 and ITGB2 were significantly upregulated in IBD tissues, consistent with the results of GSE207022. We then performed experiments to identify the hub genes STAT1, CCR5 and ITGB2 significantly upregulated in IBD. STAT1, as the first STAT family member, is a major interferon (IFN)‐signalling component involved in several cellular functions in response to cytokines and growth factors.^39^, ^40^ Studies have reported that STAT1 can transcriptionally regulate LCP2 and TNFAIP2 and that p‐STAT1 increases H3K27ac enrichment at the LCP2 and TNFAIP2 loci through epigenetic modification, contributing to the development of IBD.^16^ Another study reported that the bioactive substance *Lycium barbarum* polysaccharide showed protective effects in the intestinal mucosa by regulating macrophage polarization mediated by the STAT1 and STAT6 pathways.^41^ CCR5 is upregulated in IBD,^42^ and CCR5 blockade ameliorates inflammation in mice with IBD.^43^ However, the role of ITGB2 in the pathogenesis of IBD has not been studied.

Integrin subunit beta 2 is one subunit of the β2 integrins, which was reported to involve an inflammatory reaction in diseases such as acute lung injury,^44^ cardiac hypertrophy^45^ and keloid scar tissue.^46^ Moreover, ITGB2 silencing reduces CRC metastasis by regulating EMT.^20^ In this study, we confirmed the expression of ITGB2 in vivo and evaluated the role of ITGB2 in IBD both in vivo and in vitro IBD models. First, IBD mouse models were established by DSS treatment, and the three upregulated hub genes (STAT1, CCR5 and ITGB2) were analysed using qPCR and WB blotting. Consistent with the bioinformatic analysis results, STAT1, CCR5 and ITGB2 were significantly upregulated in the IBD mouse model. In addition, we used an in vivo ITGB2 siRNA to interfere with ITGB2 expression in an IBD mouse model and found that ITGB2 silencing alleviated the symptoms of IBD. Bioinformatic analysis showed that ITGB2 was positively associated with resting DCs. However, the mechanism through which ITGB2 regulates resting DCs in IBD remains unclear and requires further investigation. Generally, patients with IBD are more likely to develop CRC.^47^, ^48^ IBD‐associated CRC develops under chronic inflammation conditions, and pro‐inflammatory immune cells and their products, such as inflammatory factors and chemokines, contribute to cancer development.^47^ Therefore, investigation of the anti‐inflammatory or immunosuppressive mechanisms that may be involved in the development of CRC in patients with IBD specifies the selection of anti‐inflammatory or immunosuppressive agents used in these patients. Our study indicates that ITGB2 is significantly upregulated in several cancers, consistent with previous reports.^49^, ^50^ Studies have reported that LINC01272 promotes EMT and metastasis by regulating miR‐876/ITGB2 in CRC, indicating that ITGB2 is involved in tumour metastasis in CRC.^20^ However, the exact role of ITGB2 has not yet been validated in vivo. In addition, the role and mechanism of action of ITGB2 in CRC remain unclear. This study was performed in vitro and in vivo to evaluate the role of ITGB2 in CRC. Silencing ITGB2 significantly decreased cell proliferation and suppressed tumour growth, indicating that ITGB2 is a tumour‐promoting gene in CRC. Moreover, ITGB2 was upregulated in CAC tissues compared with paracancerous tissues. The risk of CRC in patients with IBD increases significantly, mainly because of the pro‐tumour effects of chronic intestinal inflammation.^48^, ^51^ Therefore, improved control of mucosal inflammation during CRC surveillance is effective. In our study, silencing ITGB2 could effectively attenuate inflammation, which may indicate that ITGB2 may be a therapeutic target for IBD and CAC treatment in the future.

In conclusion, our study provides insights into the landscape of immune cell infiltration in IBD and the immune cellular molecular mechanisms of IBD. In vivo and in vitro experiments demonstrated that ITGB2 was significantly upregulated in LPS‐induced cells and DSS‐treated tissues, and in vivo, siRNA of ITGB2 had therapeutic effects in an IBD mouse model. Silencing of ITGB2 decreased cell proliferation and tumour growth in vitro and in vivo, which may provide a new therapeutic approach for IBD or CAC.

## AUTHOR CONTRIBUTIONS

**Rong Xu:** Conceptualization (equal); data curation (equal); formal analysis (equal); writing – original draft (equal). **Wei Du:** Visualization (equal); writing – review and editing (equal). **Qinglong Yang:** Investigation (equal); methodology (equal). **Ashuai Du:** Funding acquisition (lead).

## FUNDING INFORMATION

This study was supported by grants from The Key Project of Scientific Research Plan of Hunan Provincial Health Commission (C202301047982, Wei Du), The Wings Scientific and Technological Foundation of The First People's Hospital of Changde City (2022ZZ05, Wei Du; 2024ZC02, Rong Xu) and The Fundamental Research Funds for Science and Technology Foundation of Guizhou Province (ZK[2024]‐463, Qinglong Yang).

## CONFLICT OF INTEREST STATEMENT

There are no conflicts to declare.

## Supporting information


Data S1.

## Data Availability

Data supporting the findings of this study are available in the article and supplementary material.
