# Supplementary material for: ITGB2 related to immune cell infiltration as a potential therapeutic target of inflammatory bowel disease using bioinformatics and functional research
Source: J Cell Mol Med. 2024 Aug 1;28(15):e18501. doi: 10.1111/jcmm.18501 (PMC11293422; doi:10.1111/jcmm.18501)
Supplement: Supplementary file 1 — Data S1. [file JCMM-28-e18501-s001.docx]

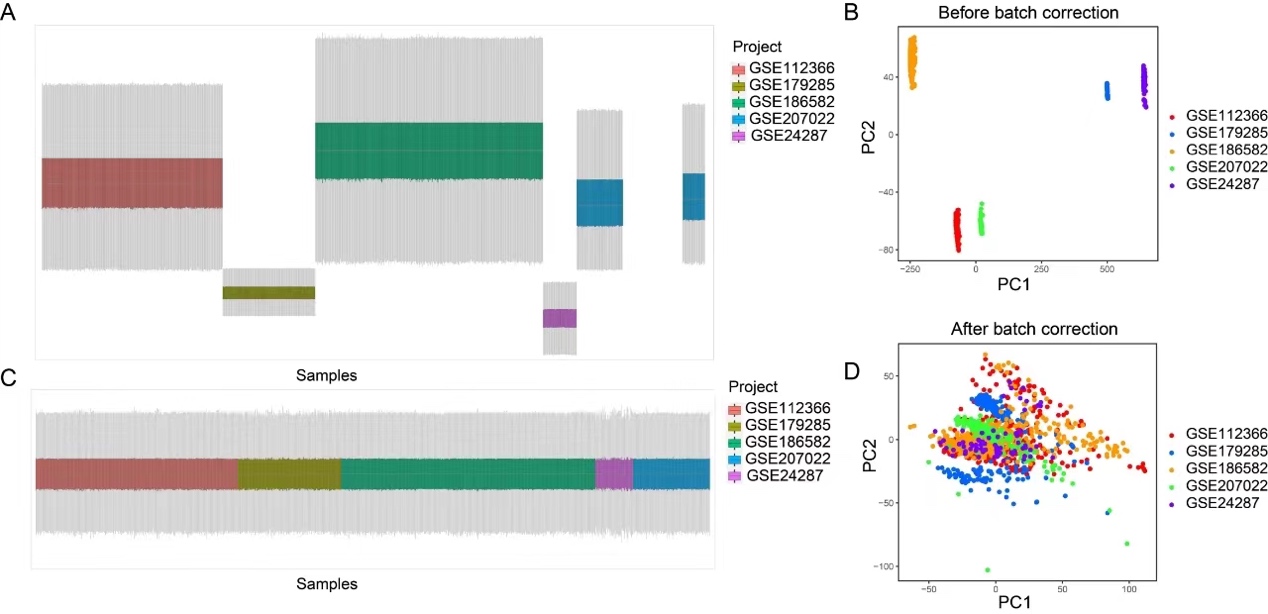


**Figure S1 Data normalizing.** (A) The data before (A-B) and after (C-D) batch correction was visualized in box plot and principal component map.


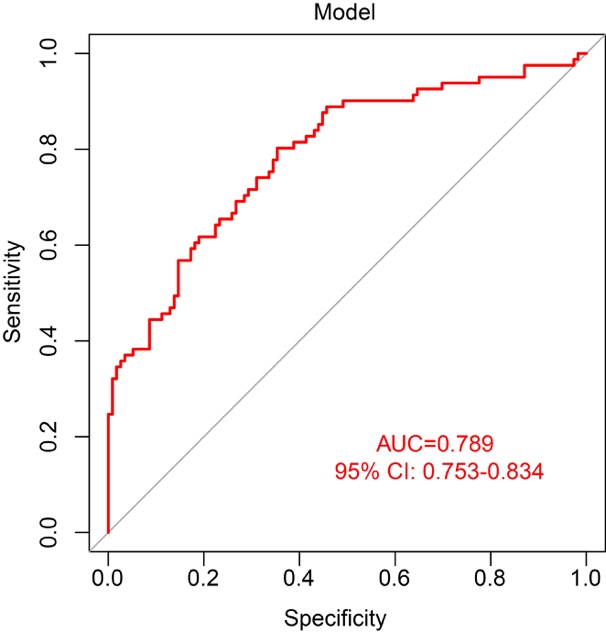


**Figure S2 The diagnostic ROC curve of STAT1 and ITGB2 combined indicates its good diagnostic performance.**


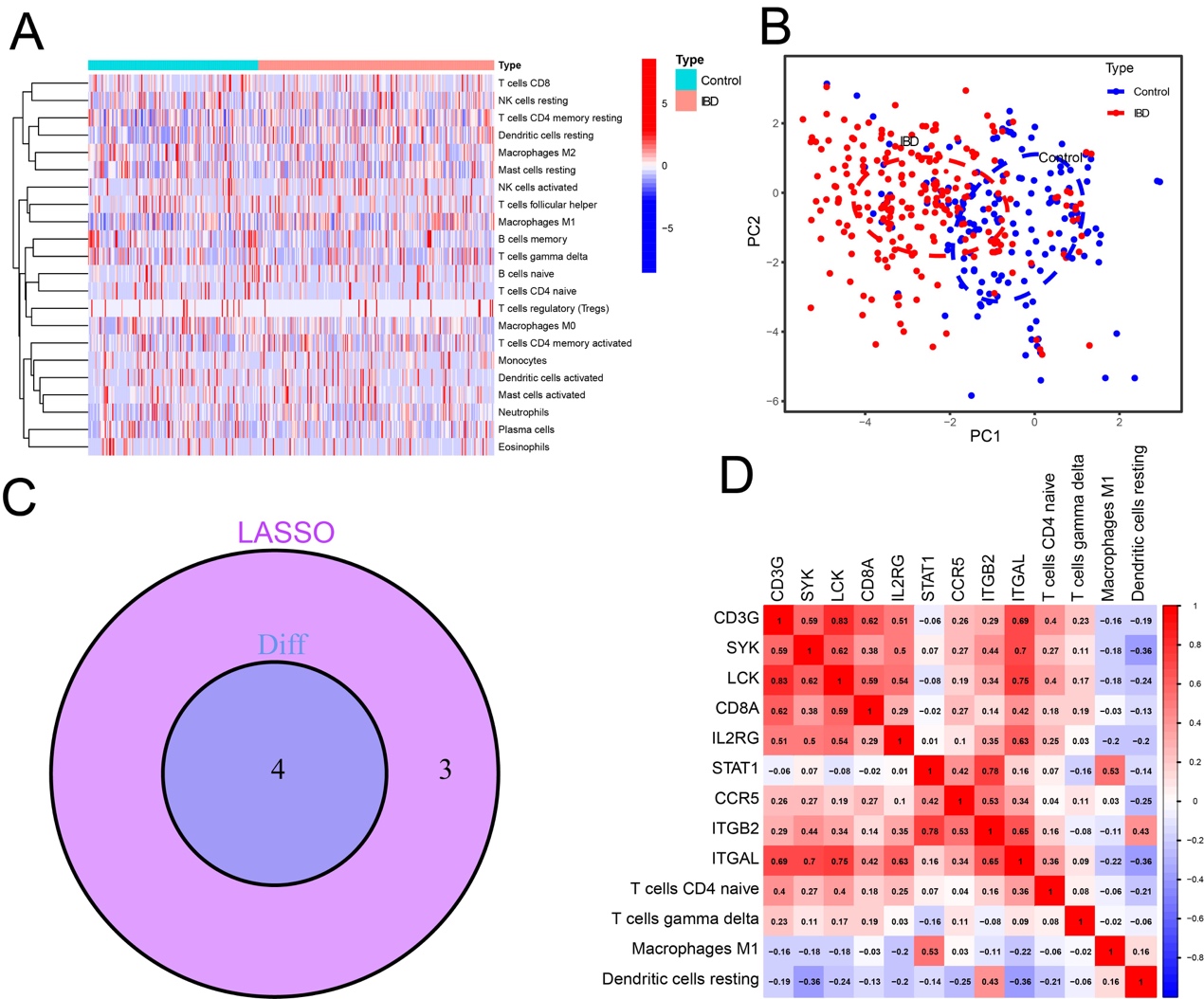


**Figure S3 Immune cell infiltration in IBD and control tissues.**

1. Heatmap showed the 22 types of immune cells.
2. PCA analysis was used to categorize the infiltrating immune cells between IBD and control tissues.
3. Vennen was used to extract the difference immune cells.
4. The correlation analysis between hub genes and immune cells.


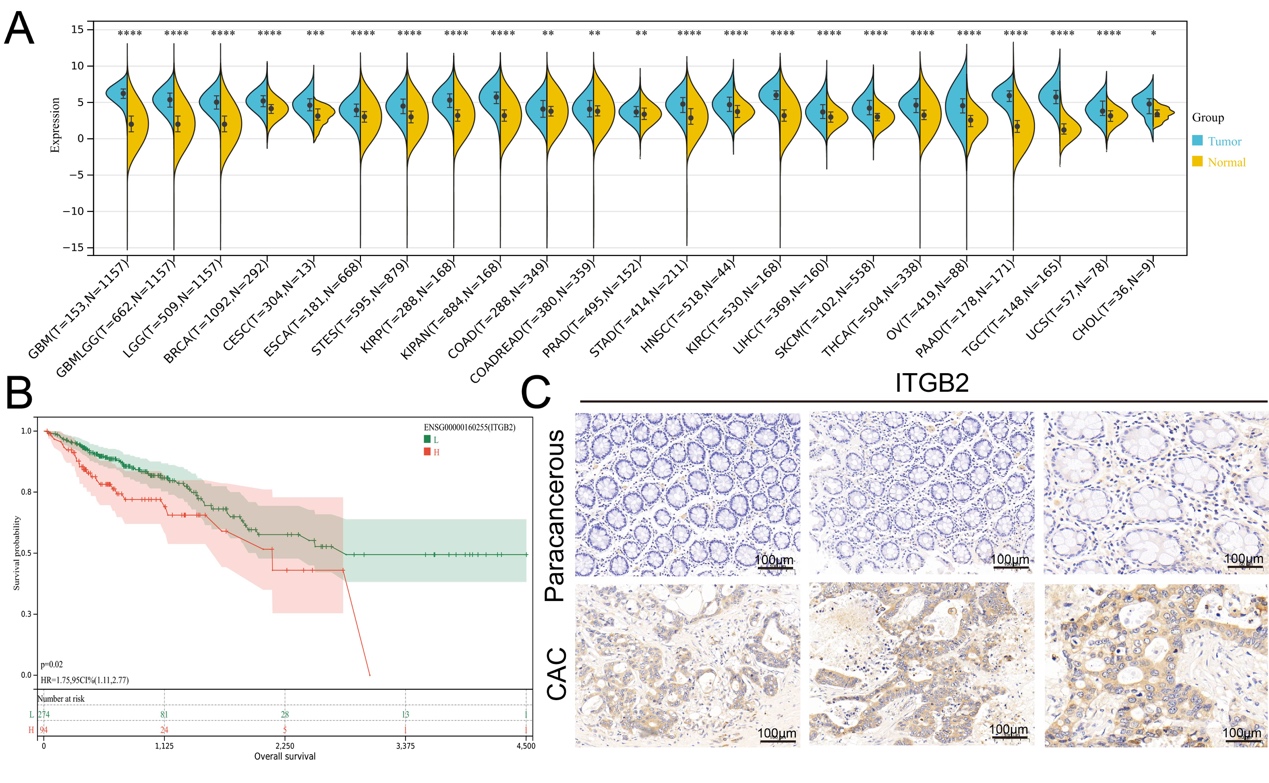
**Figure S4 The expression of ITGB2 in CRC**

1. The expression of ITGB2 in pan-cancers.
2. High expression of ITGB2 in CRC was positively related to poor prognosis.
3. IHC was used to detect the ITGB2 protein level in CRC tissues.

**Table S1.** Table of primers

| Primer sequences | Forward primer | Revers primer |
| --- | --- | --- |
| ITGB2 | 5′ ATGTAAGTGGCCGTCCTTGG 3′ | 5′ GGAAGCCGTCACTTTGAGGA 3′ |

**Table S2.** Correlation analysis for clinicopathologic parameters about ITGB2 expression in colorectal cancer patients

|  | N=40 | | ITGB2 | | | | | |
| --- | --- | --- | --- | --- | --- | --- | --- | --- |
|  |  |  | low | | high | | ꭕ^2^ | *p* |
| Age | |  | |  | |  | 2.699 | 0.1 |
| <50 | 23 | | 12 | | 11 | |  |  |
| >50 | 17 | | 14 | | 3 | |  |  |
| Gender | |  | |  | |  | 0.115 | 0.736 |
| Male | 27 | | 14 | | 13 | |  |  |
| Female | 13 | | 6 | | 7 | |  |  |
| TNM stage | |  | |  | |  | 4.552 | 0.033 |
| ǁ/ǁ | 14 | | 10 | | 5 | |  |  |
| III/IV | 26 | | 8 | | 17 | |  |  |
| Expression of ki67(%) | |  | |  | |  | 0.406 | 0.524 |
| >50 | 26 | | 14 | | 12 | |  |  |
| <50 | 14 | | 9 | | 5 | |  |  |
| CEA (ng/ml) |  | |  | |  | | 0.852 | 0.356 |
| >2.06 | 19 | | 10 | | 9 | |  |  |
| <2.06 | 21 | | 8 | | 13 | |  |  |
